# Hybrid Modification of Wheat Bran Using Microbial Processing and Ultrasound: Enhancements in Bran Composition and Bread Quality †

**DOI:** 10.3390/foods14020167

**Published:** 2025-01-08

**Authors:** Esra Sik, Hatice Bekiroglu, Necattin Cihat Icyer, Gorkem Ozulku

**Affiliations:** 1 Food Technology, Vocational School, Halic University, Alibeykoy Campus, Istanbul 34060, Turkey; esrask@halic.edu.tr; 2 Food Engineering Department, Faculty of Chemical and Metallurgical Engineering, Yildiz Technical University, Davutpasa Campus, Istanbul 34220, Turkey; bkroglu@yildiz.edu.tr; 3 Food Engineering Department, Faculty of Agriculture, Sirnak University, Mehmet Emin Acar Campus, Sirnak 73000, Turkey; 4 Food Engineering Department, Faculty of Engineering and Architecture, Mus Alparslan University, Mus 49250, Turkey; n.icyer@alparslan.edu.tr

**Keywords:** wheat bran modification, optimization, bread characteristics, nutritional properties

## Abstract

This study investigates the effects of microbial bioprocessing (MB), ultrasound treatment (UT), and their combined application (hybrid method, HM) on the functional and nutritional enhancement of wheat bran (WB) and its impact on bread quality. MB was performed by using *Saccharomyces cerevisiae* with *Levilactobacillus brevis* LABE 32 (MB32) and *Lactiplantibacillus plantarum* LABE 29 (MB29). MB32 significantly increased soluble dietary fiber (SDF) and reduced phytic acid content by up to 25.7% when compared to the control. UT further decreased phytic acid content by 52.2% and enhanced phenolic compound release, contributing to improved antioxidant activity. The hybrid method (HM) demonstrated the strongest effect, reducing phytic acid content by 95% and enhancing antioxidant properties, including a 2.4-fold increase in bound antioxidant activity (bound-AA). Bread produced from modified WB showed improvements in specific volume (SV), texture, and nutritional composition. The HM-treated WB yielded bread with the highest SV, approximately 10% greater than the control, while MB29 produced significantly harder bread than other samples (*p* < 0.05). The HM-treated bread had the highest crust L* value and softest texture (*p* < 0.05). Nutritionally, only UT and HM treatments significantly increased the total dietary fiber (TDF) content, with the most pronounced increase observed in the HM treatment. Phytic acid degradation in the WB modified with MB32 and UT was in accordance with their breads, notably lowering phytic acid content. Additionally, MB32 and HM increased total phenolic content (TPC) and antioxidant activity, enhancing the bread’s overall nutritional quality. In conclusion, the hybrid application of MB and UT (HM) proved to be the most effective in improving the functional and nutritional properties of WB and the resulting bread, including increased dietary fiber content, reduced phytic acid levels, and enhanced antioxidant activity.

## 1. Introduction

Wheat bran (WB), which constitutes approximately 22–25% of wheat grain by weight, is a valuable by-product of the milling process due to its high content of dietary fibers (DFs), vitamins, minerals, and bioactive compounds [1]. Consumption of DFs in the diet is associated with a reduced risk of several diseases, including obesity, diabetes, cardiovascular disease, and colon cancer [2].

DFs are found in cereal bran in two forms: soluble (SDF) and insoluble (IDF) [3]. Studies suggest that SDF is particularly effective in reducing blood cholesterol levels, while IDF plays a critical role in preventing colon cancer. Moreover, SDF helps control blood glucose by increasing food viscosity, thereby slowing glucose absorption. SDF also influences the texture, structure, and viscosity of foods [4]. For adults, the recommended daily intake of DF typically ranges between 30 and 35 g for men and 25 and 32 g for women [5]. Nevertheless, the *EFSA Journal*, which contains the scientific outputs of the European Food Safety Authority, also reports that daily DF consumption is below the recommended value of 25 g/day [6]. WB consists of 55–60% DF. Despite being a good source of DF, these nutritional components are largely removed during the refined flour production [2].

Currently, various methods are employed to enhance the dietary fiber content of food products, each offering distinct characteristics and advantages. A widely used approach is the addition of β-glucan, primarily sourced from oats and barley, which is recognized for its health benefits, such as lowering blood cholesterol levels and improving postprandial blood glucose and insulin responses. However, its incorporation can influence sensory properties, such as thickness and flavor, though low-molecular-weight β-glucan minimizes these effects [7]. Another method involves utilizing by-products from cereals, fruits, vegetables, and algae, which are rich in dietary fiber. These by-products not only increase fiber content but also enhance hydration, oil-holding capacity, viscosity, texture, sensory attributes, and shelf-life while being low in calories, cholesterol, and fat [8].

Therefore, WB incorporation into food formulations such as bakery products has been one of the concerns of food science in recent years. However, some drawbacks of using WB in bread-making have been revealed by many studies such as the reduction in bread volume and negative effects on texture, color, and taste [9,10,11]. Consequently, modifying WB to retain its nutritional benefits while minimizing its adverse effects on bread quality has become a growing area of interest. Techniques to modify WB include physical, chemical, and biological methods, each aiming to enhance the fiber’s functionality and reduce its negative impacts on food products [12].

Microbial bioprocessing (MB) is a biological method that is used to modify WB. During MB, the lactic acid bacteria (LAB) and yeasts involved in fermentation produce acidity, which makes the bran’s cell wall more accessible to carbohydrate-degrading enzymes. This process leads to alterations in the nutritional characteristics (such as soluble dietary fiber and antioxidant activity) and technological properties (hydration and viscosity) of the bran [12,13,14]. MB treatment also releases active compounds in WB, and new bioactive substances can be generated during fermentation [15]. MB has been highlighted as a cost-effective method due to its ability to utilize low-cost raw material such as wheat bran—a globally abundant by-product—as a substrate [16]. Furthermore, MB is considered environmentally friendly, as it consumes less energy compared to other modification techniques such as chemical, thermal, or high-energy processes [17]. However, alternative methods can be associated with some limitations: Thermal methods, while improving storage stability and functional properties, often reduce vitamin content and negatively affect sensory attributes [18,19]. Chemical methods such as alkaline and acid treatment can cause harmful residues and decrease phenolic content and antioxidant activity [20]. Physical modification methods, e.g., hydrothermal treatment of WB, have disadvantages such as a low treatment capacity, high pressure requirements, and uneven heating. In addition, reducing the particle size so it is too small has a negative effect on the bread [21]. In the view of cost-effectiveness and environmental sustainability compared with techniques that either involve higher operating costs or greater environmental impacts, MB seems to be a competitive and promising approach for the modification of WB due to these limitations in alternative methods.

The ultrasound technique (UT), as a non-thermal application, has also been used for extracting phenolic compounds and dietary fibers from WB or inactivating some enzymes such as peroxidase and lipoxygenase [22]. Ultrasound technology harnesses the physical, chemical, and biochemical effects of sound waves above humans’ audible range (>20 kHz) within a molecular environment. It is widely utilized in the food industry for various applications, including freezing, thawing, drying, extraction, sterilization, and the inactivation of microbes and enzymes [23]. Yet, to our knowledge, there are no studies that suggest the use of UT for modifying cereal bran.

This study aims to enhance the nutritional and functional properties of WB by developing a hybrid technique combining MP and UT. Also, MB and UT were studied individually to modify WB and compared with a control. In addition, the effects of those treatments on the nutritional and quality characteristics of breads containing WB were also investigated.

## 2. Materials and Methods

### 2.1. Materials

WB samples were produced by milling wheat of the Tekirdag cultivar (harvested in June 2022), obtained from the Trakya Agricultural Research Institute, Edirne, Turkey. Milling was performed with a laboratory-type milling machine (Prodigy 2, Tekpa, Antalya, Turkey) with an efficiency of 66–67%. The coarse WB obtained had a particle size of approximately 900 µm. The proximate compositions of the WB were 12.15 ± 0.93 g/100 g moisture, 4.69 ± 0.33 g/100 g fat, 15.10 ± 1.38 g/100 g protein, 44.29 ± 0.31 g/100 g dietary fiber, and 4.66 ± 0.24 g/100 g ash.

The microbial bioprocessing (MB) was performed using two LAB, namely Levilactobacillus brevis LABE 32 and Lactiplantibacillus plantarum LABE 29, and a Saccharomyces cerevisiae TGM 55. They were obtained from the sourdough culture collection of Yildiz Technical University, Food Engineering Department.

The dietary fiber assay kit was from Sigma-Aldrich (TDF100A, Sigma-Aldrich Inc., Bornem, Belgium). The enzymatic procedure was used to determine phytic acid content (K-PHYT, Megazyme, Wicklow, Ireland). The medium used for the cultivation of LAB (MRS agar and MRS broth) and yeast (Sabouraud dextrose agar and Sabouraud dextrose broth) was supplied from Merck (Darmstadt, Germany). All other chemicals and reagents were of analytical grade.

The refined flour (13.24% moisture, 11.5% protein, 10.4% gluten, and 60.1% water absorption) used in the bread-making was supplied commercially (Söke flour, Aydın, Turkiye). The non-iodized refined salt (Billur, İzmir, Turkiye) and instant dry yeast (Pakmaya, İzmit, Turkiye) were purchased from local markets.

### 2.2. Methods

#### 2.2.1. Ultrasound Treatment (UT)

WB suspensions were prepared at a ratio of 1.0 g of WB to 7.5 mL of deionized water in 250 mL beakers. The suspensions were ultrasonically treated (Hielscher UIP1000-1000W-20 kHz, Teltow, Germany) with a 22 mm diameter probe (Hielscher sonotrode BS4D22) coupled with a flow cell (Hielscher FC100L1K-1S). The ultrasound device operates at a frequency of 24 kHz with an output power of 400 W, with a range of amplitude from 20 to 100%.

##### Experimental Design

The experimental design was carried out using Design Expert software, version 13.0.5.0 (State-Ease Inc., Minneapolis, MN, USA). The level ranges of the experimental conditions were selected based on a review of the literature, and the independent variables were defined in preliminary experiments. The goal of studies using ultrasound on WB has primarily been the extraction of various components. However, its use for modifying WB has not been observed.

A full factorial experimental design was applied in this study. For this design, amplitude level (20, 40, 60, 80, and 100%) and processing time (5, 10, and 15 min) were set as independent variables for the ultrasound treatment (UT) process. This design included 15 experimental points with seven dependent variables—suspension viscosity, FPC (free phenolic content), BPC (bound phenolic content), release of phenolic compounds (%), free-AA (free antioxidant activity), bound-AA (bound antioxidant activity), and release of AA (%)—measured as responses to examine the effects of the independent variables. Each trial was conducted in triplicate to ensure accuracy. Table 1 lists the experimental points and corresponding response values. Verification analyses were performed under the optimum process conditions obtained after the optimization process.

#### 2.2.2. Microbial Bioprocessing (MB)

##### Activation of Bacteria

*Lacp. plantarum* LABE 29 and *Levl. brevis* LABE 32 were cultivated in anaerobic conditions on MRS agar at 37 °C for 48 h while S. cerevisiae TGM 55 was cultivated on Sabouraud dextrose agar at 30 °C for 48 h. Single colonies on agar were inoculated into the appropriate liquid media for each microorganism (MRS broth for LAB and Sabouraud dextrose broth for yeast). After approximately 12 h, the cells were recovered by centrifugation, resuspended in sterile purified water, and washed twice under sterile conditions. In summary, the fermentation suspension was prepared by combining the LAB and yeast suspensions in equal volumes (1:1, *v*/*v*).

##### Fermentation

Wheat bran and sterile deionized water were mixed at a ratio of 1.0 g/7.5 mL and the cultures were incorporated into the mixture with a final cell concentration of ca. 8 log cfu g^−1^ for LAB and 7.5 log cfu g^−1^ for yeast. The following combinations were used:

MB32: Levl. brevis LABE 32 and S. cerevisiae TGM 55.

MB29: Lacp. plantarum LABE 29 and S. cerevisiae TGM 55.

Fermentation was carried out at 30 °C for 24 h. Following the fermentation, WB samples were dried at 40 °C for further analyses until reaching a final moisture content of ~14%.

#### 2.2.3. Hybrid Modification (HM)

In this modification step, wheat bran samples modified under optimal ultrasonic conditions were then treated with MB using a bacterial combination that shows promising results in modifying DF, reducing phytic acid, and increasing suspension viscosity.

#### 2.2.4. Determination of Functional Properties of WB

For total water-retention capacity (TWRC), a suspension containing WB/water (1:7.5) was centrifuged (Hettich, UNIVERSAL 320 R) for 10 min at 4000× *g*. After separation of the supernatant, the filtrates were dried overnight at 90 °C [24]. TWRC was calculated according to the following equation:

(1) $$TWRC\left(\frac{mL}{g}\right)=\frac{M_{C}-M_{D}}{M_{D}}$$

M_C_: pellet mass after centrifugation.

M_D_: pellet mass after drying.

To determine the strong water-retention capacity (SWRC) of wheat bran, 50 mg of bran was weighed with ±0.01 mg precision, mixed with 700 µL deionized water, centrifuged at 15,000× *g* for 10 min, and dried at 90 °C overnight to measure the insoluble dry matter [25]. SWRC was computed according to the following equation:

(2) $$SWRC(\frac{mL}{g})=\frac{m_{centr}-m_{dry}-m_{column}\times m_{filter}}{m_{dry}}$$

m_centr_: mass of material in column after centrifugation.

m_dry_: mass of material in the column after drying.

m_column_: mass of the column.

m_filter_: the body of water retained by the filter.

The steady-state viscosity of bran suspensions was measured using a rotational rheometer (Anton Paar, MCR 302) following the adapted methodology of De Bondt et al. (2020), where 25 mL of sample was stabilized at measurement temperature and stirred to prevent sedimentation, and the viscosity was determined by increasing the shear rate from 10 to 100 s^−1^.

#### 2.2.5. Bread-Making Procedure

Bread production followed the AACCI standard method No: 10-10B [26], using 15% wheat bran (WB) incorporated into refined flour. Five types of bread were produced, coded as CB, MB29B, MB32B, UTB, and HMB. Control bread (CB) was made using untreated WB. Microbial-bioprocessed WB with a combination of *Lacp. plantarum* LABE 29/*S. cerevisiae* TGM 55 and *Levl. brevis* LABE 32/*S. cerevisiae* TGM 55 was used to produce MB29B and MB32B, respectively. The bread with ultrasound-treated WB was named UTB. The ultrasound-treated WB which had subsequently modified by the microbial bioprocess was used to produce the hybrid-modified bread (HMB). Water absorption was determined by farinograph analysis to obtain a final dough consistency of 500 BU. The dough, containing 2 g of dry yeast, 1.5 g of salt, and water based on farinograph results, was kneaded using a mixer (Öztiryakiler, Istanbul, Turkiye) and fermented in a cabinet (Nuve TK 252, Ankara, Turkiye) at 85% relative humidity and 30 °C in 30 min intervals (as first and second punching). After punching, the dough was panned and fermented for 55 min (30 °C, 85% RH) and then baked at 235 °C for 25 min in a stone-bottom oven (Fimak, Konya, Turkiye).

#### 2.2.6. Bread Analyses

Bread volume was measured using the rapeseed displacement method (AACC, 1990), and specific volume was calculated by dividing the bread volume by its weight, with the mean value taken from two samples per formulation.

The textural properties of bread samples were assessed using a texture analyzer (SMS TA.XT2 Plus, Glasgow, UK) with a 36 mm aluminum probe and a 5 kg load cell. Two slices, each 1.25 cm thick, were tested by applying 50% compression at a speed of 5.0 mm/s, measuring the force (N). Hardness, springiness, cohesiveness, and chewiness were calculated by the texture exponent connect software, with four measurements taken for each bread type.

The color values of the bread samples were measured using a chromameter (CR-400 Konica Minolta Sensing, Inc., Osaka, Japan), assessing lightness (L*), redness (a*), and yellowness (b*). The ΔE value was calculated according to Equation (3). Four replicate measurements were taken for both crust and crumb.

(3) $$\Delta E=\sqrt{{\left(L-L_{ref}\right)}^{2}+{\left(a-a_{ref}\right)}^{2}+{\left(b-b_{ref}\right)}^{2}}$$

#### 2.2.7. Chemical Analyses of WB and Bread Samples

Wheat bran and bread samples were defatted prior to the extraction of free and bound phenolics to determine the total phenolic content (TPC) and antioxidant capacity. The extraction of free and bound phenolics from the samples was conducted following the methodology described by Shamanin et al. [27]. For the extraction of free phenolics, 0.5 g of defatted samples was mixed with 5 mL of acetone/water (1:1) and shaken in a water bath (25 °C, 200 rpm, 1 h). After centrifugation (2500× *g*, 4 °C, 10 min), supernatants were collected from two additional extractions and stored at 4 °C. The remaining pellet was dried at 30 °C overnight to extract bound phenolics. Solvent removal was performed via rotary evaporation (Hei-VAP Advantage, Heidolph, Schwabach, Germany) at 40 °C, 60 rpm, and 65 mbar, and the extract was dissolved in 2 mL of methanol and stored in amber vials at −18 °C. The dried pellet was hydrolyzed with 20 mL of 2.0 N NaOH for 4 h at 200 rpm, neutralized with 6 M HCl, and washed with 10 mL of hexane, with vortex-mixing and centrifugation (4000× *g*, 10 min). This step was repeated five times to remove free fatty acids and then bound phenolics were extracted.

The TPC of the extracts was determined spectrophotometrically in accordance with the methodology outlined by Singleton and Rossi [28]. The antioxidant capacity of the extracts was determined by means of a DPPH (2,2-Diphenyl-1-picrylhydrazyl) assay [29].

The total dietary fiber (TDF), soluble dietary fiber (SDF), and insoluble dietary fiber (IDF) contents of wheat bran and bread samples were analyzed following the AACCI Method No: 32-07.01 using the enzymatic–gravimetric approach [30]. Additionally, the phytic acid content was determined according to AOAC Method No: 986.11 [31]. All results were given on a dry-matter basis.

#### 2.2.8. Statistical Analysis

The significance of the main effects and interactions of variables was assessed using ANOVA, with a *p*-value threshold of 0.05 for significance. Model fit was determined using the R^2^ coefficient and Fisher’s F-test through Design Expert V. 13.0.5.0, while the figures were generated using OpenAI’s ChatGPT artificial intelligence model. Further analyses were performed using SPSS version 24.0, and significant differences among the different WB and bread samples were identified through Duncan’s multiple range tests (*p* < 0.05).

## 3. Results

### 3.1. Optimization and Verification Analysis of Ultrasound Treatment (UT) Conditions

**Figure 1:**
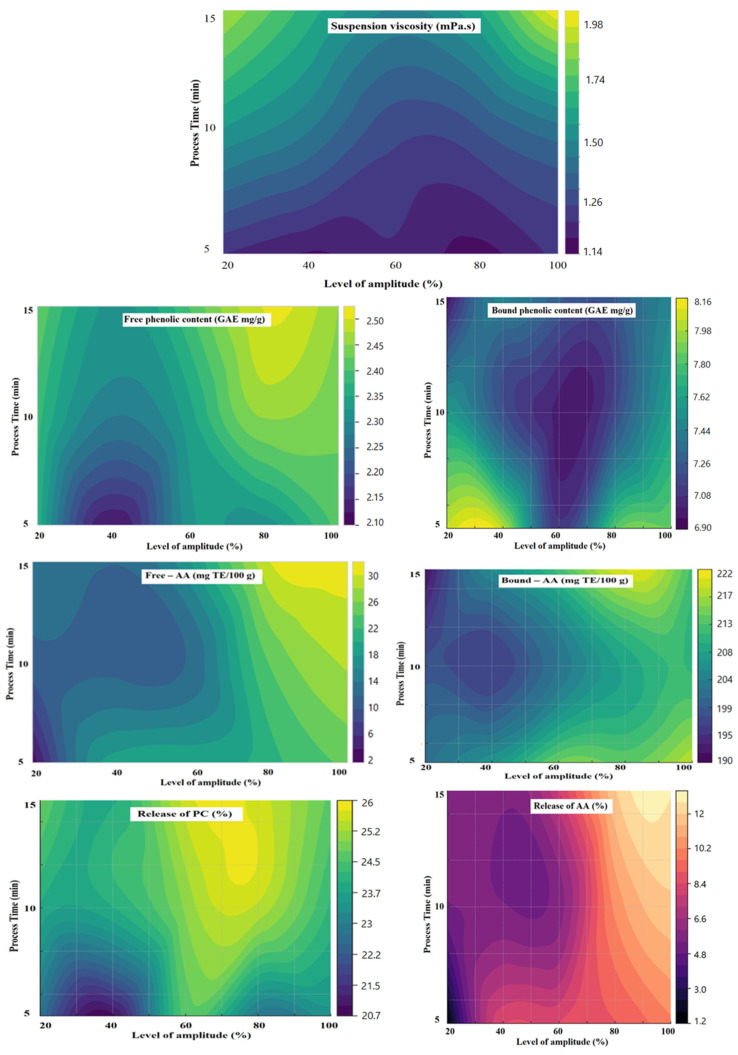
Contour plots illustrating the interactions between responses and independent variables.

Table 2 shows the effects of the independent variables (processing time and amplitude level) on the dependent variables (suspension viscosity, FPC, BPC, release of phenolic compounds, free-AA, bound-AA, and release of AA) during the ultrasonic modification of wheat bran. In this study, a significance level of 0.05 was selected. *p*-values below this threshold indicate that the model can adequately explain the variation in the respective response. An R^2^ value close to 1 suggests that the model provides a good fit for the data. Figure 1 shows the contour plots illustrating the interactions between responses and independent variables.

There is a statistically significant increase in suspension viscosity as both processing time and ultrasound intensity increase. The increase in processing conditions enhances the soluble fiber content, which in turn raises the viscosity. For this dependent variable, the R^2^ value was determined to be 0.9137.

The free phenolic content (FPC) was found to be significantly influenced by both independent variables. Lower process times and lower ultrasound intensities resulted in a low FPC level. Process times of 10 min or more and ultrasound intensities of 60% or higher led to an increase in FPC values. Ultrasonication is known to enhance food quality parameters by facilitating the extraction of phenolic compounds from plant materials. Under the applied processing conditions, some of the phenolic compounds in wheat bran (WB) were released. The highest FPC values were obtained under conditions of high processing time and high ultrasound intensity. For this dependent variable, the R^2^ value was 0.8843.

For bound phenolic content (BPC), both independent variables also had a statistically significant effect. Ultrasound intensity, up to 60%, led to a reduction in phenolic compound content, potentially due to cavitation effects causing the degradation of phenolic compounds during extraction. Some phenolic compounds were released, while others may have degraded. When ultrasound intensity exceeded 60%, the extraction of phenolic compounds increased; however, as processing time increased, the extraction rate decreased. While an extension of processing time led to a decrease in phenolic compound content, after 10 min, the effect of processing time diminished. The highest BPC values were obtained with a low processing time and high ultrasound intensity. The R^2^ value for this dependent variable was 0.8956. Similarly to FPC, the extension of processing time increased the quantity of free phenolic compounds. Thus, high processing time and high ultrasound intensity promoted the release of FPC in WB modification.

The release of PCs (%) represents the percentage of free PCs (FPC) released after ultrasonic treatment relative to the total PC content (free + bound). In the experimental design, both independent variables were found to have statistically significant effects on this value, with an R^2^ value of 0.8655 for this dependent variable.

For free-AA, process time was found to have no statistically significant effect, while ultrasound intensity had a statistically significant effect. The highest free-AA was observed with high processing time and high ultrasound intensity, while the lowest free-AA content was obtained under conditions of low processing time and low ultrasound intensity. Under these treatment conditions, some of the antioxidant compounds in WB were released. The R^2^ value for this dependent variable was 0.8436.

Similarly, for bound-AA content, processing time showed no statistically significant effect, whereas ultrasound intensity had a statistically significant effect. The highest total-AA content was observed with a high processing time and high ultrasound intensity, while the lowest total-AA content was achieved with a processing time of 10 min and low ultrasound intensity. The R^2^ value for this dependent variable was 0.8432.

The value for the release of AAs (%) is the percentage of AA released after ultrasonic treatment according to the total. In the experimental design, ultrasonic intensity was statistically significant for this variable, whereas processing time was not. The R^2^ value for this dependent variable was also 0.8655.

The optimization of data obtained from factorial experimental design was performed by selecting the “maximize” option for the FPC, release of PCs, free-AA, and release of AAs responses. The other responses were set as “in range”. All process parameters and responses were assigned equal importance with a weight of “3”. The model identified the optimal ultrasound treatment (UT) conditions to maximize all responses as 100% amplitude and set a processing time of 15 min. Since the desirability value was determined to be 0.933, which is close to 1, verification analyses of the data obtained at the optimum point were conducted. The process conditions with the highest desirability, the responses, and the verification analysis results at the optimum conditions are presented in Table 3. The experimental analysis results at the optimum point were highly consistent with the optimum values predicted by the experimental design. Therefore, it can be concluded that the model is sufficiently accurate in predicting behavior under the given UT conditions.

### 3.2. Effects of Modification Techniques on WB Characteristics

#### 3.2.1. Ultrasound Treatment (UT)

**Figure 2:**
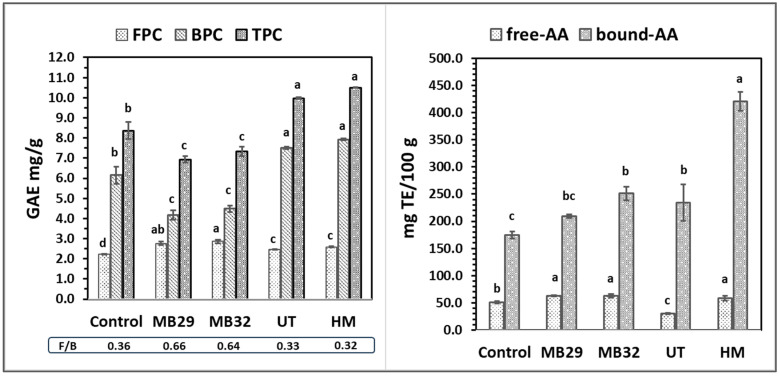
Phenolic contents and antioxidant activities of control and modified WB. Different lowercase show significant differences between samples (*p* < 0.05). Control: untreated wheat bran; MB29: microbial bioprocess with *Lc. plantarum* LABE29 and *S. cerevisiae* TGM55; MB32: microbial bioprocess with *Levl. brevis* LABE32 and *S. cerevisiae* TGM55; UT: ultrasound treatment; HM: hybrid modification; F/B: sum of free to sum of bound ratio.

Table 4 and Figure 2 present the functional, nutritional, and antioxidant properties of the samples modified by UT.

UT applied to wheat bran has frequently been investigated for the extraction of functional components such as phenolic compounds and xylans [32,33,34], in addition to its ability to enhance stability [35].

Total water-retention capacity (TWRC), strong water-retention capacity (SWRC), and suspension viscosity are considered as functional properties in cereal bran. SWRC is associated with water retained in nanopores, while TWRC is attributed to water held within micropores and is defined as the amount of water retained by hydrated fibers after the application of external forces, such as pressure or centrifugation [24,36].

In this study, UT increased the TWRC, SWRC, and suspension viscosity of WB (Table 4). However, no significant increments in TWRC and viscosity were observed when compared to the control sample. UT caused a higher SWRC than other treatments and the control sample (*p* < 0.05). The SWRC value is associated with the surface area of WB [37,38]. The high intensity of UT (100% amplitude) likely induced a significant increase in surface area, allowing bran cells to retain larger amounts of water at the nanoscale. As a result, UT may reduce particle size [39] and enhance water retention by increasing water-binding sites through the separation of fiber and protein [40].

The TDF content was increased by UT due to the rise in SDF content when compared to the control (Table 4). It was reported that UTs can modify the composition, structure, and characteristics of SDF polysaccharides extracted from by-products such as peels and other vegetables [41]. In this study, UT may have increased the extraction of SDFs, which were not efficiently detected in the control sample.

UT effectively reduced the phytic acid content of wheat bran by 52.2%, showing its role in promoting phytic acid degradation (Table 4). Some studies have investigated the effect of ultrasonic treatment on the phytic acid content of some cereal bran and reported phytic acid reductions of 17% for oat and 38% for barley bran [42].

Figure 2 shows the changes in phenolic content and antioxidant activity of wheat bran modified by UT. A significant increase in both FPC and BPC, compared to untreated bran, led to a 19.2% rise in TPC. In a study of Habuš et al. [22], a 15 min application of high-intensity UT (80% amplitude) resulted in an 11% increase in total phenolics. Phenolic compounds are typically found as soluble conjugates or in insoluble forms bound to cell wall structures. Therefore, ultrasound-assisted hydrolysis is widely studied for releasing these bound phenolics. In this study, UT was directly applied to the WB suspension for modification, followed by standard phenolic determination methods. The UT likely improved phenolic detection by increasing the accessibility of these compounds. As a result of UT, free-AA decreased while bound-AA increased. This may be attributed to the destructive impact of UT on the activity of phenolic compounds already accessible in untreated bran. However, UT enhances AA overall compared to the control, as it improved the accessibility of bound phenolics. To better understand these results, further analysis is required to examine the changes in phenolic acid composition, which may contribute to the antioxidant activity of WB.

#### 3.2.2. Microbial Bioprocessing (MB)

MB was applied at 30 °C for 24 h, with the resulting cell numbers shown in Table 5. In the MB performed using *Levl. brevis* LABE 32 (MB32), LAB and yeast showed microbial growth of 1.30 and 1.23 log cfu/mL after 24 h, respectively. These values were 2.38 log cfu/mL for LAB and 1.44 log cfu/mL for yeast in the MB performed using *Lacp. plantarum* LABE 29 (MB29). Additionally, a significant pH reduction was observed in both microbial combinations during fermentation, with the increase in acidity being more pronounced in the MB32 combination. Prückler and Lorenz [43] examined the impact of different LAB species, including *Lacp. plantarum* and *Levl. brevis*, on the complex matrix of wheat bran. Their fermentation procedure resulted in a 0.85 log cfu/g increase in cell count of *Levl. brevis* and a 0.94 log cfu/g increase in *Lacp. plantarum*. Similarly, our study also showed that MB29 was more effective than MB32 in promoting LAB growth (Table 5).

The functional and nutritional properties of the untreated and modified wheat bran samples are presented in Table 4. The TWRC values of the bran increased in both microbial combinations compared to the control, whereas the SWRC values decreased following MB treatment. This phenomenon may be attributed to the enlargement of micropores caused by the aggregation of wheat bran fibers as a result of the fermentative process. Zhao et al. reported an increase in TWRC values in their study, where they fermented wheat bran using yeast and lactic acid bacteria to enhance its properties [44]. The suspension viscosity, which is related to the water-soluble components of wheat bran [45] and provides insight into the amount of bran to be added to bran-enriched products [46], increased after MB treatment, with a more pronounced effect observed in the MB32 combination.

One of the key aims of WB modification is to lead the conversion of IDF into SDF since SDF exhibits superior physiological benefits compared to its insoluble counterpart [47]. MB treatment increased the SDF content and decreased the IDF content. The increment of SDF was more pronounced for MB32 treatment. However, MB treatment caused no significant change in TDF content (Table 4). A study that investigated the modification of wheat bran with yeast and LAB revealed that fermentation led to up to a 2-fold increase in SDF content, whether microorganisms were used individually or in combination [44]. The fermentation of WB enhances the production of SDF, with each microbial strain exhibiting varying degrees of effectiveness in the conversion of IDF to SDF [48].

Table 4 also presents the effects of MB treatments on the phytic acid content in WB. The MB29 combination resulted in a 12.1% reduction in phytic acid content compared to the control, while the MB32 combination achieved a more substantial reduction of 25.7%. These findings align with various studies that have demonstrated the role of LAB in reducing phytic acid content [44,49].

The phenolic content and antioxidant activities of both untreated and modified WB were shown in Figure 2. The MB29 and MB32 treatments increased the FPC values by 24.3% and 28.8%, respectively. Nevertheless, both microbial combinations led to a more pronounced decrease in BPC values, ultimately resulting in an overall reduction in TPC following the MB treatment when compared to the control. Similarly, the reduction in TPC after fermentation of WB was also observed by Zhao and Guo [44]. However, the ratio between free and bound phenolics was higher in the MB-treated samples than the control in this study (Figure 2). This was explained in a study of Spaggiari et al. [49] as the metabolized conjugated phenolic compounds by LAB, thus breaking the linkage of the phenolics bound to the cell wall polysaccharides. MB treatments improved the antioxidant activity (AA) of bran in both free-AA and bound-AA fractions. This enhancement was statistically significant for bound-AA in the MB32 treatment.

#### 3.2.3. Hybrid Modification (HM)

The HM step aimed to combine the MB and UT techniques, which were previously studied separately on bran, to enhance the modifying effect and achieve greater improvements in bran properties. WB processed under optimal UT conditions was then treated with the MB32 microbial combination. MB32 was chosen over MB29 in the HM step due to its more effective modification of dietary fiber composition, its superior reduction in phytic acid, and its greater increase in antioxidant activity, as discussed earlier (Table 4 and Figure 2).

**Figure 3:**
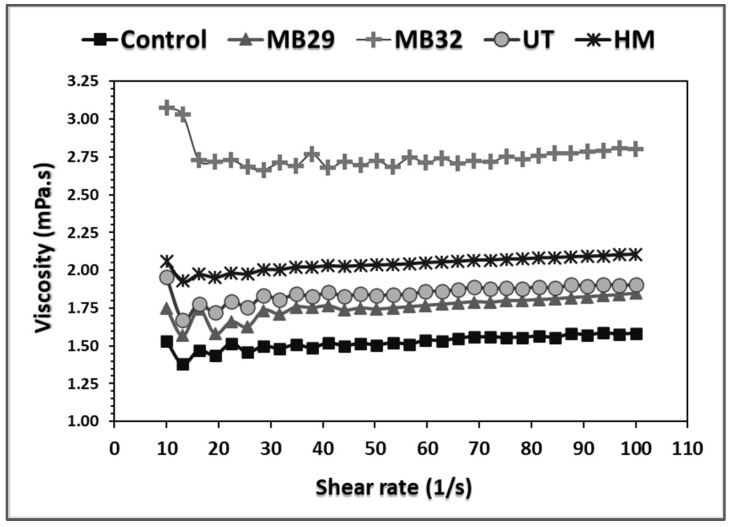
Typical suspension viscosity of WB. Control: untreated wheat bran; MB29: microbial bioprocess with *Lc. plantarum* LABE29 and *S. cerevisiae* TGM55; MB32: microbial bioprocess with *Levl. brevis* LABE32 and *S. cerevisiae* TGM55; UT: ultrasound treatment; HM: hybrid modification.

When examining the effects of the treatments on the hydration properties of wheat bran, no significant differences were found between the HM, UT, and MB32 applications in terms of TWRC. All treatments increased total water-retention capacity (Table 4). Nevertheless, the SWRC results varied across the treatments. While UT significantly increased SWRC, the value returned to that of the untreated sample following the subsequent MB32 treatment. The most significant increase in the suspension viscosity of WB was observed with the MB32 treatment, followed by HM and UT. Viscosity provides insight into the amount of water soluble fractions. As shown in Table 4, there is a clear correlation between SDF content and viscosity. Consistent with MB32 producing the highest suspension viscosity (Figure 3), this treatment also resulted in the highest SDF value. While MB treatment, regardless of the microbial combination, did not result in a significant change in TDF content, both UT and HM treatments led to a notable increase in TDF compared to the control bran. However, the difference between UT and HM treatments was not statistically significant. All modification treatments resulted in significant degradation of phytic acid, with the HM treatment being the most effective, achieving a 95% reduction compared to control bran. This was followed by UT and MB32 treatments, which reduced phytic acid by 52.2% and 25.7%, respectively.

As illustrated in Figure 2, the MB32 treatment contributed to an increase in FPC, whereas UT and HM treatments had a negative impact on FPC. In contrast, UT and HM treatments positively affected BPC, and consequently TPC, leading to a significant increase. The underlying reasons for these outcomes have been discussed in previous sections addressing MB and UT individually. However, the hybrid technique does not appear to offer any advantage over the individual application of ultrasound in enhancing the phenolic content of wheat bran. The HM treatment had a pronounced effect on the antioxidant properties of bran, significantly increasing both free-AA and bound-AA values. In particular, the bound-AA of HM-treated bran was approximately 2.4 times higher than that of control bran, compared to 1.4 times for MB32 and 1.3 times for UT. For free-AA, MB32 and HM treatments were more effective than UT, as UT actually caused a decrease in free-AA compared to the untreated bran (Figure 2).

### 3.3. Effects of Modification Techniques on Bread Characteristics

#### 3.3.1. Bread Quality

**Figure 4:**
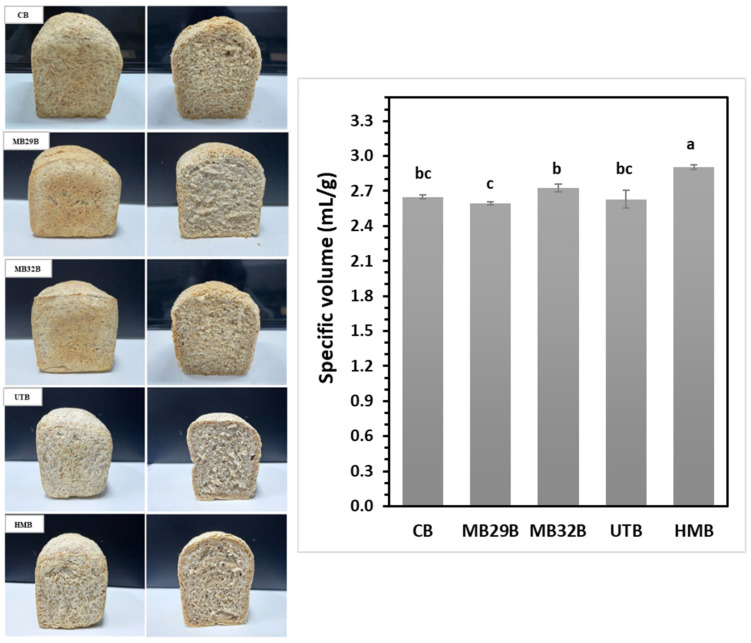
Bread images and specific volumes. Different lowercase show significant differences between samples (*p* < 0.05).

Figure 4 shows the images of breads produced with both untreated and modified WB, along with their respective specific volumes (SVs). The SV of CB (reference bread produced with untreated WB), MB29B (bread produced by using WB treated with MB29), MB32B (bread produced by using WB treated with MB32), UTB (bread produced by using WB with UT), and HMB (bread produced by using WB with the HM) were measured as 2.65 ± 0.01, 2.59 ± 0.03, 2.73 ± 0.03, 2.63 ± 0.08, and 2.92 ± 0.02 mL/g, respectively. The SV of HMB was approximately 10% higher than that of CB, making it the bread with the highest SV (*p* < 0.05). MB32B exhibited a higher SV compared to MB29B. This can be attributed to the greater effectiveness of MB32 treatment in reducing the IDF content of the bran (Table 4). According to the some studies, insoluble dietary fibers interfere with gluten network formation, thereby negatively impacting bread volume [50,51]. Furthermore, the higher TWRC of bran in the MB29 treatment (Table 4) led to greater water retention during bread production, resulting in denser, heavier breads. This, in turn, reduced the SV on a per-unit basis, making MB29B the treatment with the lowest volume. Contrary to some studies, which show that yeast and LAB fermentation significantly improve bread volumes [52,53], no significant differences were observed in terms of the SV of MB breads when compared to the CB (Figure 4).

The textural properties of the bread samples, including hardness, springiness, cohesiveness, and chewiness, are presented in Table 6. MB29B, with its lower SV (Figure 4), showed a higher hardness value due to the negative correlation between hardness and SV [54]. HMB had the lowest hardness value but was not significantly different from CB and MB32B (*p* > 0.05). Likewise, there were no significant differences in springiness and cohesiveness among the samples. In line with our findings, some studies have reported varying trends in hardness and springiness [55] as well as cohesiveness [56]. Chewiness was higher in the UTB and MB29B samples, consistent with the hardness results.

The incorporation of WB imparts a darker and more brownish hue to the final product [57], which negatively impacts consumer acceptance [21]. The color values of breads were determined as L* (0, black; 100, white), a* (−100, green; +100, red), and b* (−100, blue; +100, yellow) and are shown in Table 7. HMB and UTB showed the highest crust L* values (*p* < 0.05), indicating the lightest color among the breads, followed by other samples. While the MB technique had no effect on the crust L* value (*p* > 0.05), the ultrasound technique and hybrid modification resulted in a higher crust L* (*p* < 0.05) when compared to the control. This can be due to the ultrasound effect on the WB pigments inactivating polyphenol oxidase [22]. MB treatments had no impact on the crust a* value of the breads (*p* > 0.05), regardless of the microbial combination. However, UT and HM treatments reduced the a* value, lowering the redness level. The ΔE value measures overall color difference perceived by the human eye: ΔE < 1 is imperceptible, 1 < ΔE < 3 is subtle, and ΔE > 3 is noticeable [58]. MB29B and MB32B breads exhibited similar ΔE values, while the highest ΔE was observed in HMB, followed by UTB. As shown in Figure 4, HMB displayed the most significant color change, which is also perceivable by the human eye. However, this color trend in the crust may not fully reflect the color of the bread crumb. Among the samples, MB32B exhibited the least crumb color change compared to CB (ΔE = 4.08 ± 0.50), while HMB had the highest crumb color change (Table 7).

#### 3.3.2. Nutritional Properties of Breads

Table 8 shows the dietary fiber and phytic acid content of bread samples. The dietary fiber composition of untreated bran and bran modified by various techniques exhibited a consistent trend in the dietary fiber composition of the breads made from these bran (Table 4). Although all modification processes increased the TDF content of the breads, the most significant increases in both IDF and TDF were observed in the HMB treatment. While studies have investigated the impact of WB bioprocessing with various microbial strains on bread quality (e.g., texture, volume) [59,60], no research has been found that specifically evaluates the effect of MB on the dietary fiber composition of bread. The results of our study clearly demonstrated that the MB technique, UT, and their combined application significantly altered the dietary fiber composition of the final product. Notably, SDF content increased 2-fold in M32B and 1.9-fold in HMB compared to CB, leading to a significant increase in TDF content in the breads (Table 8).

The degradation of phytic acid in the breads were closely in line with the reductions observed in the wheat bran (Table 4). Specifically, phytic acid content in the breads decreased by 53.0%, 41.6%, 22.9%, and 16.9% for HMB, UTB, M32B, and M29B, respectively, compared to the CB. This outcome can be attributed to the phytic acid breakdown during dough fermentation by *S. cerevisiae* [61]. This dual contribution—dough fermentation and the use of modified wheat bran—was highly effective in minimizing phytic acid content.

**Figure 5:**
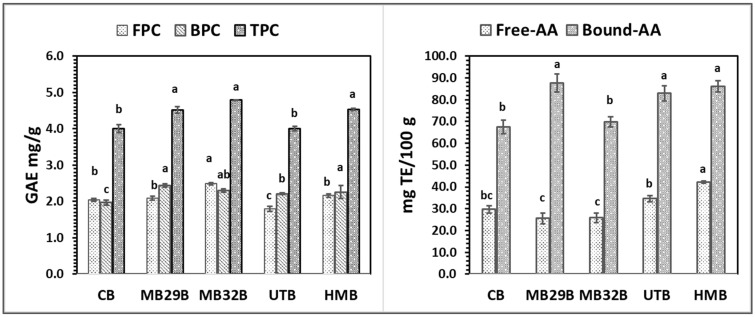
Phenolic contents and antioxidant activities of breads. Different lowercase show significant differences between samples (*p* < 0.05). CB: reference bread produced with control bran; MB29B: bread produced using bran treated with the MB29; MB32B: bread produced using bran treated with the MB32; UTB: bread produced using bran treated with the UT; HMB: bread produced using bran treated with the HM.

Figure 5 presents the TPC and antioxidant activity of free and bound phenolics of the various bread samples. The highest FPC value was observed in MB32B (2.49 ± 0.03 mg GAE/g), reflecting an increase of approximately 22% compared to CB. No significant differences were observed in terms of TPC values of MB32B, MB29B, and HMB. Bioprocessing facilitates the breakdown of starch, non-starch polysaccharides, and proteins, resulting in increased levels of reducing sugars, soluble dietary fiber, peptides, and amino acids. This process also promotes the release of insoluble phenolic compounds, which are covalently bound to cell wall polysaccharides, ultimately leading to a higher content of extractable phenolics [62]. The increase in TPC values, along with the corresponding antioxidant activities (free-AA and bound-AA), can be attributed to the use of WB treated by MB in our study. The enhanced release of phenolic compounds during bioprocessing likely contributed to these higher values, reflecting improved antioxidant potential in the breads produced (MB29B, MB32B, and HMB). In contrast, using WB with UT significantly reduced the FPC in the bread (UTB), resulting in an 11.8% lower FPC than that of the bread made with untreated bran.

The findings reveal that the free-AA and bound-AA values of CB were 29.7 ± 1.6 and 67.54 ± 3.17 mg TE/100 g, respectively, with bound phenolics contributing more significantly to antioxidant activity, similar to the bran samples (Figure 2). While the FPC value for UTB was the lowest, its free-AA surpassed that of breads modified with MB. Previous studies have also reported the reducing effect of ultrasound technology on free phenolic content [63]. It is possible that UT facilitated the emergence of phenolic acids with higher antioxidant capacity, which were better preserved during bread production. This could explain the enhanced antioxidant activity in the final product. In summary, HMB exhibited the highest total antioxidant activity, with a 32% increase compared to CB, followed by UTB (21% increase) and MB29B (16% increase).

## 4. Conclusions

This study demonstrates that the application of microbial bioprocessing (MB), ultrasound treatment (UT), and their combination (HM) significantly improves the functional, nutritional, and technological properties of wheat bran (WB) and the corresponding bread. The HM was more effective in reducing phytic acid, increasing soluble dietary fiber (SDF), and enhancing the antioxidant properties of WB. Specifically, the HM treatment achieved a 95% reduction in phytic acid, the highest bound antioxidant activity (bound-AA), and significant improvements in the bread’s specific volume and crumb hardness.

The results also showed that UT and HM treatments were the only methods that led to a significant increase in total dietary fiber (TDF) content in the final bread product, with the most pronounced increase observed in HM-treated bread.

From a quality perspective, the incorporation of modified WB positively influenced the color and texture of the bread. Breads produced with HM-treated WB exhibited the softest texture and the lightest crust color.

In conclusion, the combined application of MB and UT techniques (HM) provides the most comprehensive improvement in wheat bran and bread quality, making it a promising approach for enhancing the health benefits and consumer appeal of wheat bran-enriched bakery products. While the findings highlight the effectiveness of the hybrid modification technique, further studies are required to evaluate its cost-effectiveness and feasibility for large-scale production. Additionally, long-term health impact studies on factors such as the bioavailability of nutritional compounds and prebiotic effects are needed to assess the safety and broader implications of consuming HM-modified bread. Future studies may investigate the applicability of these modification techniques on other cereal grains and bakery products by optimizing effective parameters. This will probably ensure the wider usage of these valuable by-products.

## Figures and Tables

**Table 1:** Experimental parameters and observed response values in the ultrasonic treatment (UT) process.

	Independent Variables		Responses						
Run	Level of Amplitude (%)	Process Time (min)	Suspension Viscosity (mPa·s)	FPC (GAE mg/g)	BPC (GAE mg/g)	Release of Phenolic Compounds (%)	Free-AA (mg TE/100 g)	Bound-AA (mg TE/100 g)	Release of AA (%)
1	20	5	1.26	2.37	10.41	22.73	2.6	204.4	1.27
2	20	10	1.61	2.39	9.96	23.95	11.1	209.7	5.29
3	60	5	1.25	2.31	9.41	24.59	19.8	234.7	8.45
4	100	10	1.47	2.43	10.05	24.19	28.3	239.4	11.83
5	40	15	1.71	2.32	9.68	23.95	12.1	217.7	5.55
6	100	5	1.28	2.41	10.27	23.43	24.5	243.3	10.05
7	60	10	1.34	2.34	9.33	25.05	12.3	214.9	5.74
8	20	15	1.92	2.42	9.78	24.79	12.1	203.6	5.94
9	40	5	1.19	2.12	10.15	20.92	18.8	225.4	8.35
10	100	15	2.03	2.45	9.93	24.66	30.8	243.9	12.63
11	80	5	1.18	2.31	10.12	22.82	21.1	235.2	8.96
12	60	15	1.56	2.40	9.36	25.61	18.0	230.2	7.80
13	80	15	1.66	2.50	9.75	25.63	29.8	250.4	11.91
14	40	10	1.50	2.28	9.49	23.98	11.3	207.9	5.45
15	80	10	1.36	2.45	9.64	25.43	24.1	232.9	10.33

FPC: free phenolic content; BPC: bound phenolic content; Free-AA: free antioxidant activity; Bound-AA: bound antioxidant activity.

**Table 2:** ANOVA analysis results of full factorial design for ultrasonic treatment (UT) process.

**Suspension viscosity (mPa·s)**					
Source	Sum of squares	DF	Mean square	F-value	*p*-value
Model	0.8750	6	0.1458	14.12	0.0007
X_1_	0.1253	4	0.0313	3.03	0.0851
X_2_	0.7497	2	0.3749	36.29	<0.0001
Residual	0.0826	8	0.0103		
Cor total	0.9576	14			
R^2^ = 0.9137; Adjusted R^2^ = 0.8490					
**Free phenolic content (GAE mg/g)**					
Source	Sum of squares	DF	Mean square	F-value	*p*-value
Model	105,600	6	17,603.53	10.19	0.0022
X_1_	72,328.72	4	18,082.18	10.47	0.0029
X_2_	33,262.44	2	16,646.22	9.64	0.0074
Residual	13,817.51	8	1727.19		
Cor total	119,400	14			
R^2^ = 0.8843; Adjusted R^2^ = 0.7975					
**Bound phenolic content (GAE mg/g)**					
Source	Sum of squares	DF	Mean square	F-value	*p*-value
Model	1,635,000	6	272,500	11.44	0.0015
X_1_	903,500	4	225,900	9.48	0.0040
X_2_	731,500	2	365,800	15.35	0.0018
Residual	190,600	8	23,829.89		
Cor total	1,826,000	14			
R^2^ = 0.8956; Adjusted R^2^ = 0.8173					
**Release of PCs (%)**					
Source	Sum of squares	DF	Mean square	F-value	*p*-value
Model	19.47	6	3.25	8.58	0.0039
X_1_	7.94	4	1.99	5.25	0.0225
X_2_	11.53	2	5.76	15.24	0.0019
Residual	3.02	8	0.38		
Cor total	22.50	14			
R^2^ = 0.8655; Adjusted R^2^ = 0.7647					
**Free-AA (mg TE/100 g)**					
Source	Sum of squares	DF	Mean-square	F-value	*p*-value
Model	785.34	6	130.89	7.19	0.0069
X1	752.05	4	188.01	10.33	0.0030
X2	33.29	2	16.64	0.9143	0.4389
Residual	145.63	8	18.20		
Cor total	930.96	14			
R^2^ = 0.8436; Adjusted R^2^ = 0.7263					
**Bound-AA (mg TE/100 g)**					
Source	Sum of squares	DF	Mean square	F-value	*p*-value
Model	829.04	6	136.51	7.19	0.0069
X_1_	677.44	4	166.86	8.79	0.0050
X_2_	151.60	2	75.80	3.99	0.0628
Residual	151.90	8	18.99		
Cor total	970.94	14			
R^2^ = 0.8482; Adjusted R^2^ = 0.7343					
**Release of AAs (%)**					
Source	Sum of squares	DF	Mean square	F-value	*p*-value
Model	111.76	6	18.63	5.95	0.0123
X_1_	106.76	4	26.69	8.52	0.0055
X_2_	4.99	2	2.50	0.80	0.4834
Residual	25.05	8	3.13		
Cor total	136.81	14			
R^2^ = 0.8169; Adjusted R^2^ = 0.6795					

**Table 3:** Optimization of parameters and verification of experimental design.

	Process Time (min)	Level of Amplitude (%)	Optimization Values	Verification Values	Desirability
Suspension viscosity (mPa·s)	15	100	1.88	1.79 ± 0.05	0.933
FPC (GAE mg/g)			2.48	2.47 ± 0.01	
BPC (GAE mg/g)			7.49	9.98 ± 0.05	
Release of PCs (%)			24.90	24.74	
Free-AA (mg TE/100 g)			29.97	30.69 ± 0.88	
Bound-AA (mg TE/100 g)			214.83	234.02 ± 33.55	
Release of AA (%)			12.29	11.60	

**Table 4:** Some functional and nutritional properties of control and modified WB.

	TWRC (mL/g)	SWRC (mL/g)	Suspension Viscosity (mPa·s)	SDF (%)	IDF (%)	TDF (%)	Phytic Acid (g/100 g)
Control	3.82 ± 0.08 ^c^	1.152 ± 0.015 ^bc^	1.57 ± 0.05 ^c^	3.26 ± 0.09 ^d^	41.04 ± 0.21 ^ab^	44.29 ± 0.31 ^b^	2.72 ± 0.02 ^a^
MB29	5.98 ± 0.16 ^a^	1.076 ± 0.034 ^c^	1.69 ± 0.07 ^bc^	5.52 ± 0.38 ^bc^	39.64 ± 0.30 ^bc^	45.16 ± 0.69 ^b^	2.39 ± 0.05 ^b^
MB32	5.35 ± 0.21 ^ab^	1.039 ± 0.019 ^c^	2.71 ± 0.22 ^a^	6.92 ± 0.28 ^a^	38.16 ± 0.99 ^c^	45.08 ± 0.72 ^b^	2.02 ± 0.02 ^c^
UT	4.73 ± 0.66 ^bc^	1.496 ± 0.142 ^a^	1.79 ± 0.05 ^bc^	5.27 ± 0.06 ^c^	42.89 ± 1.08 ^a^	48.16 ± 1.02 ^a^	1.30 ± 0.01 ^d^
HM	5.50 ± 0.47 ^ab^	1.270 ± 0.020 ^b^	2.08 ± 0.06 ^b^	6.16 ± 0.24 ^ab^	43.08 ± 0.51 ^a^	49.23 ± 0.75 ^a^	0.13 ± 0.00 ^e^

Data are expressed as mean ± standard deviation of three replicates. Means with different lowercase letters in the same column indicate significant differences (*p* < 0.05) for treatments. Control: untreated wheat bran; MB29: microbial bioprocess with *Lc. plantarum* LABE 29 and *S. cerevisiae* TGM 55; MB32: microbial bioprocess with *Levl. brevis* LABE 32 and *S. cerevisiae* TGM 55; UT: ultrasound treatment; HM: hybrid modification.

**Table 5:** Microorganism growth in wheat bran suspensions during MB.

		0 h		6 h	24 h	
		Number of Cells (log cfu/mL)	pH	pH	Number of Cells (log cfu/mL)	pH
MB32	LAB	7.87	7.70	6.76	9.17	4.86
	Yeast	5.91			7.14	
MB29	LAB	7.03	7.92	6.65	9.41	5.14
	Yeast	5.76			7.20	

MB32: *Levl. brevis* LABE 32 and *S. cerevisiae* TGM 55; MB29: *Lacp. plantarum* LABE 29 and *S. cerevisiae* TGM 55.

**Table 6:** TPA parameters of bread samples.

Treatments	Hardness (N)	Springiness	Cohesiveness	Chewiness
CB	12.77 ± 0.63 ^bc^	0.90 ± 0.00 ^a^	0.82 ± 0.01 ^a^	9.37 ± 0.31 ^b^
MB29B	17.49 ± 0.19 ^a^	0.82 ± 0.03 ^a^	0.76 ± 0.00 ^a^	10.95 ± 0.48 ^a^
MB32B	12.68 ± 0.73 ^bc^	0.82 ± 0.01 ^a^	0.79 ± 0.01 ^a^	8.22 ± 0.52 ^bc^
UTB	15.31 ± 1.06 ^ab^	0.93 ± 0.04 ^a^	0.83 ± 0.04 ^a^	11.78 ± 0.91 ^a^
HMB	11.69 ± 0.79 ^c^	0.93 ± 0.00 ^a^	0.80 ± 0.01 ^a^	8.16 ± 1.47 ^c^

Data are expressed as mean ± standard deviation of three replicates. Means with different lowercase letters in the same column indicate significant differences (*p* < 0.05) for bread samples. CB: reference bread produced with control bran; MB29B: bread produced using bran treated with the MB29; MB32B: bread produced using bran treated with the MB32; UTB: bread produced using bran treated with the UT; HMB: bread produced using bran treated with the HM.

**Table 7:** Color parameters of bread samples.

	Crust				Crumb			
	L*	a*	b*	ΔE	L*	a*	b*	ΔE
CB	58.78 ± 0.96 ^b^	7.80 ± 1.21 ^a^	25.27 ± 2.13 ^a^		58.94 ± 1.05 ^a^	5.03 ± 0.21 ^a^	18.28 ± 0.37 ^a^	
MB29B	58.44 ± 2.49 ^b^	7.09 ± 0.36 ^a^	19.32 ± 1.08 ^bc^	6.37 ± 1.19 ^c^	57.25 ± 0.86 ^a^	4.09 ± 0.32 ^b^	13.63 ± 0.21 ^bc^	5.09 ± 0.37 ^ab^
MB32B	61.09 ± 1.62 ^b^	6.65 ± 0.89 ^a^	19.31 ± 0.94 ^bc^	6.59 ± 1.59 ^c^	59.27 ± 0.58 ^a^	4.00 ± 0.31 ^b^	14.37 ± 0.43 ^b^	4.08 ± 0.50 ^b^
UTB	67.02 ± 1.33 ^a^	4.30 ± 0.18 ^b^	20.39 ± 0.84 ^b^	10.25 ± 0.99 ^b^	57.51 ± 3.19 ^a^	4.04 ± 0.54 ^b^	13.91 ± 0.83 ^bc^	5.39 ± 1.36 ^ab^
HMB	68.08 ± 1.20 ^a^	2.66 ± 0.21 ^c^	17.06 ± 0.91 ^c^	13.46 ± 1.02 ^a^	57.56 ± 1.59 ^a^	3.19 ± 0.41 ^c^	13.13 ± 0.29 ^c^	5.81 ± 0.31 ^a^

Data are expressed as mean ± standard deviation of three replicates. Means with different lowercase letters in the same column indicate significant differences (*p* < 0.05) for bread samples. CB: reference bread produced with control bran; MB29B: bread produced using bran treated with the MB29; MB32B: bread produced using bran treated with the MB32; UTB: bread produced using bran treated with the UT; HMB: bread produced using bran treated with the HM.

**Table 8:** Dietary fiber and phytic acid content of breads.

	SDF (%)	IDF (%)	TDF (%)	Phytic Acid (mg/100 g)
CB	1.34 ± 0.03 ^c^	7.54 ± 0.16 ^c^	8.89 ± 0.13 ^d^	49.40 ± 0.24 ^a^
MB29B	2.24 ± 0.04 ^b^	7.15 ± 0.10 ^cd^	9.39 ± 0.14 ^c^	41.05 ± 0.31 ^b^
MB32B	2.67 ± 0.11 ^a^	7.04 ± 0.18 ^d^	9.72 ± 0.28 ^c^	38.08 ± 1.15 ^c^
UTB	2.08 ± 0.05 ^b^	8.87 ± 0.04 ^b^	10.95 ± 0.09 ^b^	28.86 ± 0.33 ^d^
HMB	2.55 ± 0.09 ^a^	9.63 ± 0.16 ^a^	12.17 ± 0.07 ^a^	23.21 ± 0.94 ^e^

Data are expressed as mean ± standard deviation of three replicates. Means with different lowercase letters in the same column indicate significant differences (*p* < 0.05) for bread samples. CB: reference bread produced with control bran; MB29B: bread produced using bran treated with the MB29; MB32B: bread produced using bran treated with the MB32; UTB: bread produced using bran treated with the UT; HMB: bread produced using bran treated with the HM.

## Data Availability

The original contributions presented in this study are included in the article. Further inquiries can be directed to the corresponding author.
